# ADAR1 Prevents R-loop Accumulation-Driven ATR Pathway Activation in Ovarian Cancer

**DOI:** 10.7150/jca.72108

**Published:** 2022-04-24

**Authors:** Hanwei Cui, Qian Yi, Min Tian, Hai Ting Yang, Yuteng Liang, Jie Huang, Qi Zeng, Weichao Sun, Jian Han, Jianxin Guo, Zhiying Yu, Wenlan Liu, Xiufeng Ye

**Affiliations:** 1 Central Laboratory, Shenzhen Samii Medical Center, Shenzhen, Guangdong 518118, P.R. China.; 2 The Department of Gynecology, Shenzhen Second People's Hospital/the First Affiliated Hospital of Shenzhen University Health Science Center, Shenzhen 518035, P.R. China.; 3 The Central Laboratory, Shenzhen Second People's Hospital/the First Affiliated Hospital of Shenzhen University Health Science Center, Shenzhen 518035, P.R. China.; 4 The Department of Gynecology & Obstetrics, Daping Hospital, Army Medical University, Chongqing 400042, P.R. China.; 5 Department of Physiology, School of Basic Medical Sciences, Southwest Medical University, Luzhou, China.; 6 Department of Reproductive Medicine, The First Affiliated hospital of Jinan University, Guang zhou, Guangdong, China, 510000.

**Keywords:** Ovarian Cancer, ADAR1, DNA Damage, R-Loop

## Abstract

Adenosine (A)-to-inosine (I) RNA editing is the most prevalent RNA editing mechanism, in which adenosine deaminase acting on RNA 1 (ADAR1) is a major adenosine deaminase. Increasing evidence suggests that editing dysregulation of ADAR1 plays an important role in human tumorigenesis, while the underlying mechanism remains elusive. Here, we demonstrated that ADAR1 was highly expressed in ovarian cancer tissues and negatively correlated with progression free survival of ovarian cancer patients. Importantly, silence of ADAR1 repressed ovarian cancer cell growth and colony formation *in vitro* and inhibited ovarian cancer cell tumorigenesis *in vivo*. Further cell cycle and transcriptome profile analysis revealed that silence of ADAR1 in ovarian cancer cells induced cell cycle arrest at G1/G0 stage. Mechanistically, loss of ADAR1 caused R-loop abnormal accumulation, thereby contributing to single stand DNA break and ATR pathway activation. Additionally, ADAR1 interacted with DHX9 to regulate R-loop complex formation, and A-to-I editing of nascent RNA repressed R-loop formation during co-transcriptional process. Together, our results identify a novel ADAR1/R-loop/ATR axis critical for ovarian cancer progression and a potential target for ovarian cancer therapy.

## Introduction

Ovarian cancer is the most lethal malignant tumor in gynecological cancers with a poor 5-year survival rate of only 28% in advanced stages 1. The standard treatment for ovarian cancer is cytoreductive surgery and taxane/platinum based chemotherapy. However, more than 70% of patients with advanced stages recur within 5 years and develop drug resistance after standard treatment 2. Thus, it's critical to identify more effective treatment strategies and novel molecular target agents for improving survival rates for patients with ovarian cancer.

Adenosine (A) to inosine (I) RNA editing is the most prevalent RNA editing mechanism in human, which is catalysed by double-stranded RNA (dsRNA)-specific adenosine deaminase (ADAR) protein family (ADAR1, ADAR2 and ADAR3) 3. Recent studies indicated that ADAR1 was upregulated in several cancers, including hepatocellular carcinoma 4, esophageal cancer 5, 6, leukemia 7, and lung cancer 8, 9, due to amplification of ADAR1 gene locus or activation of JAK-STAT pathway. Further studies have revealed that hyper editing of different types of RNA by ADAR1, including mRNA and non-coding RNA, contributes to the malignant phenotype, or even cancer progression 10, 11. Besides RNA editing function, other functions of ADAR1 have also been explored, including forming a complex with DICER to regulate microRNA processing 12, competing with STAUFEN to prevent mRNA-decay 13. Recently, loss of ADAR1 was reported to cause hyperactivation of PKR and translational shutdown 14, and even overcome resistance to immune checkpoint blockade treatment in a subset of cancer cell lines 15, 16.

In spite of the growing evidence for A-to-I RNA editing in tumorigenesis, the clinical significance and the functional role of ADAR1 in ovarian cancer remains unexplored. Herein, we found that ADAR1 was significantly upregulated in ovarian cancer tissue and knockdown of ADAR1 repressed ovarian cancer cell line tumorigenesis through inducing cell cycle arrest. Further mechanistic experiments revealed that repression of ADAR1 did not activate PKR and type 1 IFN pathway, but rather caused single strand DNA break and abnormal R-Loop accumulation. Our data shed new light on the role of ADAR1 in promoting carcinogenesis in ovarian cancer.

## Results

### High ADAR1 expression correlates with poor PFS in Ovarian cancer

**Figure 1:**
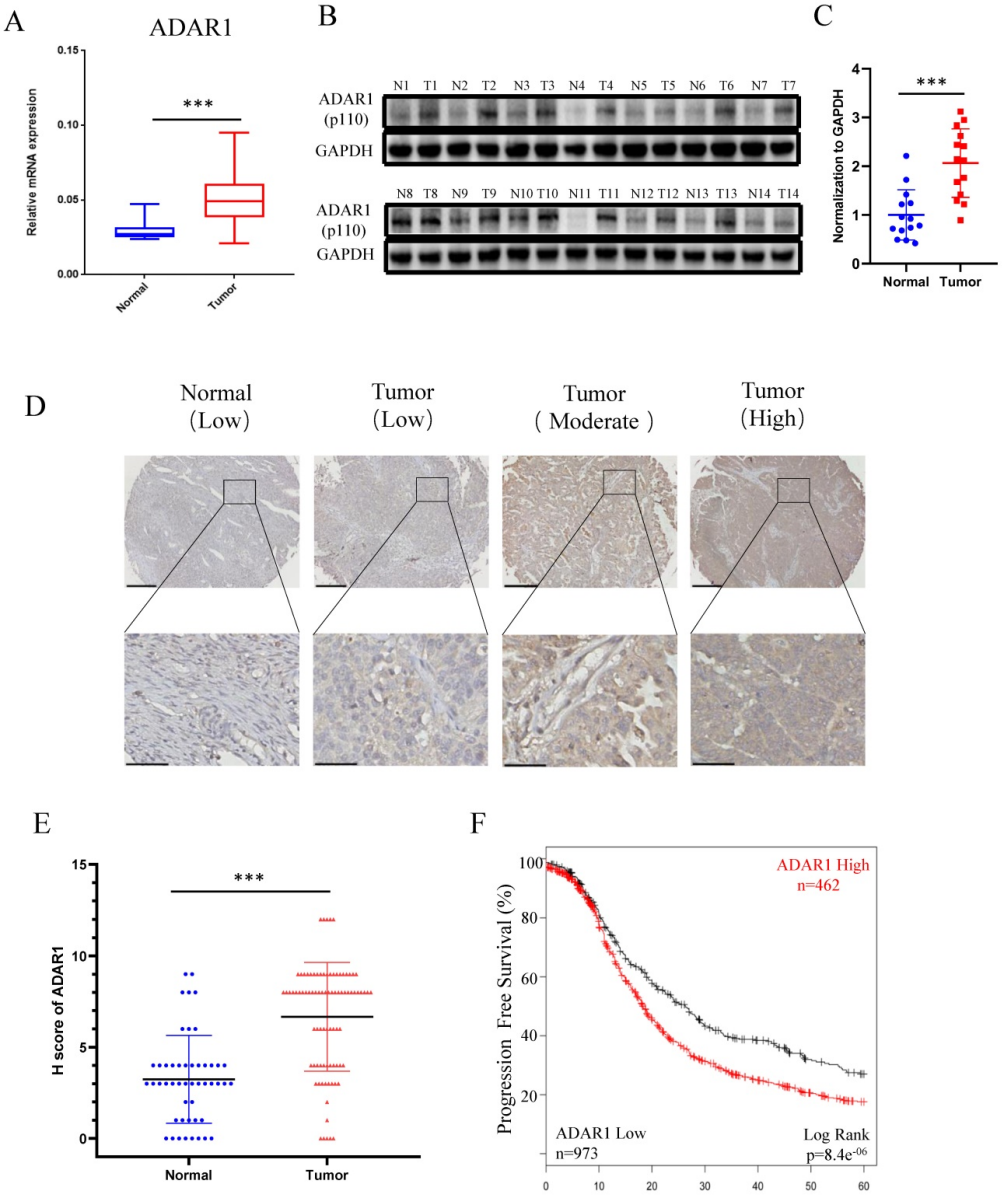
** High ADAR1 expression correlates with poor PFS in Ovarian cancer. (A)** mRNA levels of ADAR1 in ovarian cancer tissues and normal ovarian surface epithelium tissues were quantified by quantitative reverse-transcription PCR. ADAR1 expression was normalized by GAPDH expression and each of the noncancerous ovarian surface epithelium tissues were used as a control. Data were shown as mean ± SD (***, *P* < 0.001; **, *P* < 0.01; *, *P*< 0.05. **(B)** Protein levels of ADAR1 in ovarian cancer tissues (T) and normal ovarian surface epithelium tissues (N) were determined by Western blot. GAPDH expression served as endogenous reference. **(C)** Quantification analysis of ADAR1 protein levels of (B) by gray values were shown as mean ± SD (***, *P* < 001; **, *P* < 0.01; *, *P*< 0.05). **(D)** Assessment of ADAR1 protein expression in ovarian cancer tissues and normal ovarian surface epithelium tissues by immunohistochemical analysis. The representative microphotographs showed high, moderate, and low staining of ADAR1 in tissue array. Upper scale bar, 200 µm; lower scale bar, 50 µm. **(E)** Quantification analysis of ADAR1 protein stain of (D) by H scores. Data were shown as mean ± SD (***, *P* < 0.001; **, *P* < 0.01; *, *P* < 0.05). **(F)** Kaplan-Meier curve of progression-free survival of ovarian cancer patients according to the expression level of ADAR1 protein in ovarian cancer tissues (ADAR1 High-expression, n = 462 and ADAR1 Low-expression, n = 973).

To determine the status of ADAR1 in ovarian cancer clinical samples, we assessed ADAR1 mRNA expression in ovarian cancer tissues (n = 32) and normal ovarian surface epithelium tissues (n = 21) using quantitative reverse-transcription PCR (qRT-PCR). As shown in Fig. 1a, ADAR1 mRNA expression was significantly upregulated in ovarian cancer tissue compared to normal tissue (P < 0.001). Consistent with the mRNA results, Western blot analysis showed increased protein level of ADAR1 in ovarian cancer tissue (Fig. 1b, c). To further verify the upregulation of ADAR1 in ovarian cancer tissue, we generated a tissue array containing 87 ovarian cancer tissues and 49 normal ovarian surface epithelium tissues for immunohistochemistry staining. A histological evaluation based on the histo-score (H-score) showed that strong ADAR1 staining (H-score ≥ 6) was found in 61 of 87 (70.1%) ovarian cancer tissues, while weak ADAR1 staining (H-score ≤ 6) was found in 41 of 49 (83.7%) normal tissues (Fig. 1d, e). Progression free survival Kaplan-Meier analysis of published ovarian cancer microarray dataset (N = 1435) by “Kaplan-Meier Plotter (kmplot.com)” 17 showed that high ADAR1 protein level strongly correlated with a poor outcome, whereas low ADAR1 protein level was associated with good progression free survival (Fig. 1f, *P* < 0.01). Collectively, these results indicated that ADAR1 might play a critical role in the occurrence and progression of ovarian cancer.

### Loss of ADAR1 inhibits ovarian cancer cell proliferation, clonogenicity and migration

**Figure 2:**
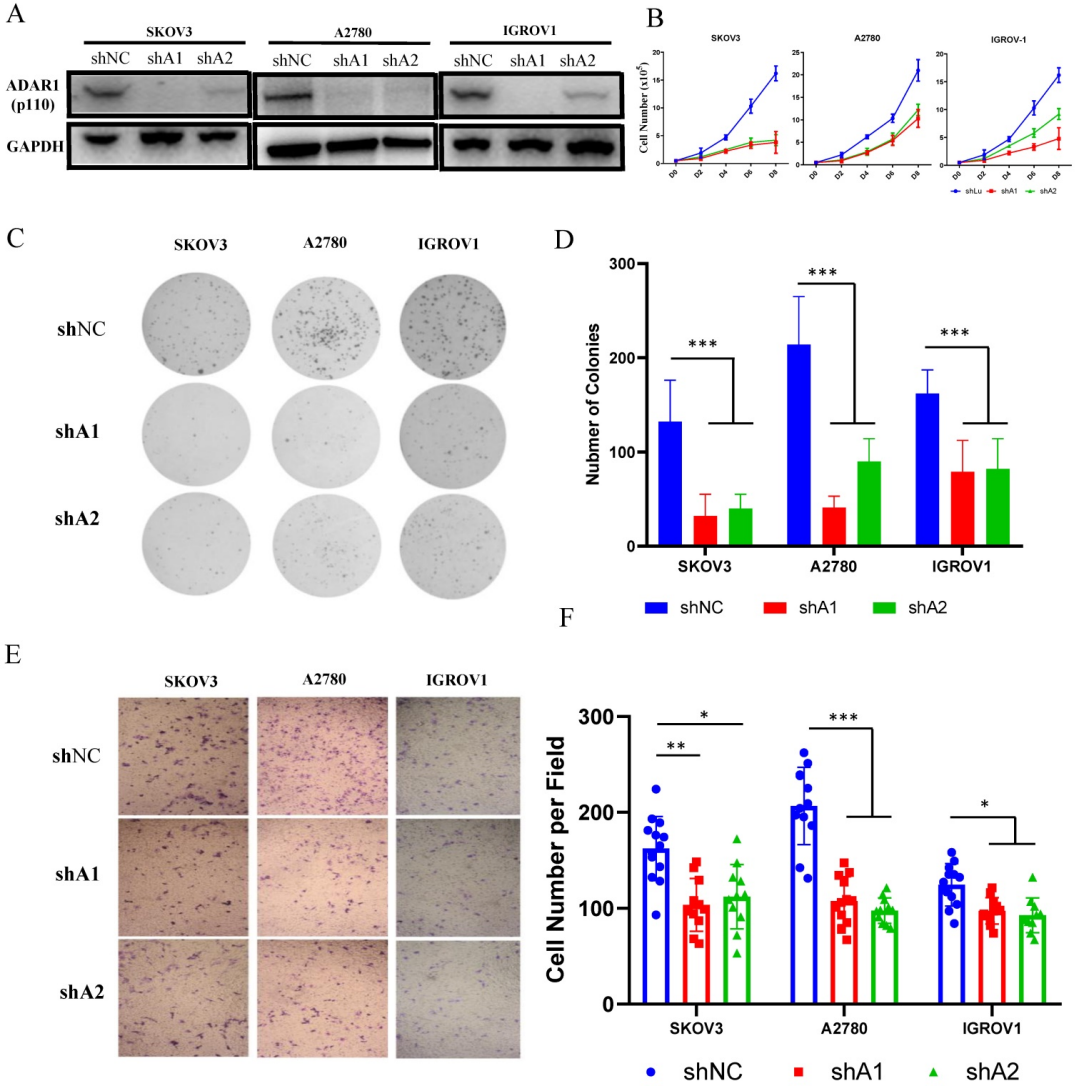
** ADAR1 deficiency inhibits ovarian cancer cell proliferation, clonogenicity and invasion *in vitro*. (A)** Protein levels of ADAR1 were determined in three ovarian cancer cell lines (SKOV3, A2780 and IGROV1) by Western blot. shA1 means shRNA 1 targeting ADAR1; shA2 means shRNA 2 targeting ADAR1. **(B)** Cell growth curve of SKOV3, A2780 and IGROV1 with or without ADAR1 silence. All data were shown as mean ± SD from four independent experiments. **(C)** The representative microphotographs showed the colony formation of SKOV3, A2780 and IGROV1 cells with or without ADAR1 silence. **(D)** The number of colonies in (C) were shown in histograms. All data were shown as mean ± SD from three independent experiments (***, *P* < 0.001; **, *P* < 0.01; *, *P* < 0.05. **(E)** The representative microphotographs showed the migration of SKOV3, A2780 and IGROV1 cells with or without ADAR1 silence. The migrated cell numbers of (E) were shown in histograms. All data were shown as means ±SD from three independent experiments (***, *P* < 0.001; **, *P* < 0.01; *, *P <* 0.05).

Since ADAR1 upregulation was associated with poor prognosis in ovarian cancer, we attempted to explore the function of ADAR1 in ovarian cancer using *in vitro* cultured cell lines. Three ovarian cancer cell lines (SKOV3, A2780 and IGROV1) were used to construct stably knockdown cell lines by short hairpin mediated RNAs (shRNAs) of two independently targeted regions of ADAR1. Western blot was performed to examine the knockdown efficiency and the results showed that both ADAR1 shRNAs (shA1 and shA2) could significantly reduce ADAR1 protein levels in all three ovarian cancer cell lines compared with non-targeted scramble shRNA (Fig. 2a). We then tested the effect of ADAR1 shRNAs on growth and clonogenicity of ovarian cancer cells. Compared with scramble shRNA, both ADAR1 shRNAs significantly inhibited the proliferation of ovarian cancer cell lines, reflected by reduced cell numbers in ADAR1 shRNAs-treated cells (Fig. 2b, P <0.01). Moreover, the ovarian cancer cell lines incubated with ADAR1 shRNAs showed smaller and fewer number of colonies than scramble shRNA-treated cells (Fig. 2c, d). These results together indicated that ADAR1 played an important role in promoting ovarian cancer cell growth and clonogenicity. Furthermore, we assessed cell migration using non-matrigel-coated transwell chambers and found that ADAR1 knockdown significantly repressed cell migration (Fig. 2 e, f), which is consistent with previous report in lung cancer cells 9.

### Loss of ADAR1 inhibits tumorigenesis of ovarian cancer cells *in vivo*

**Figure 3:**
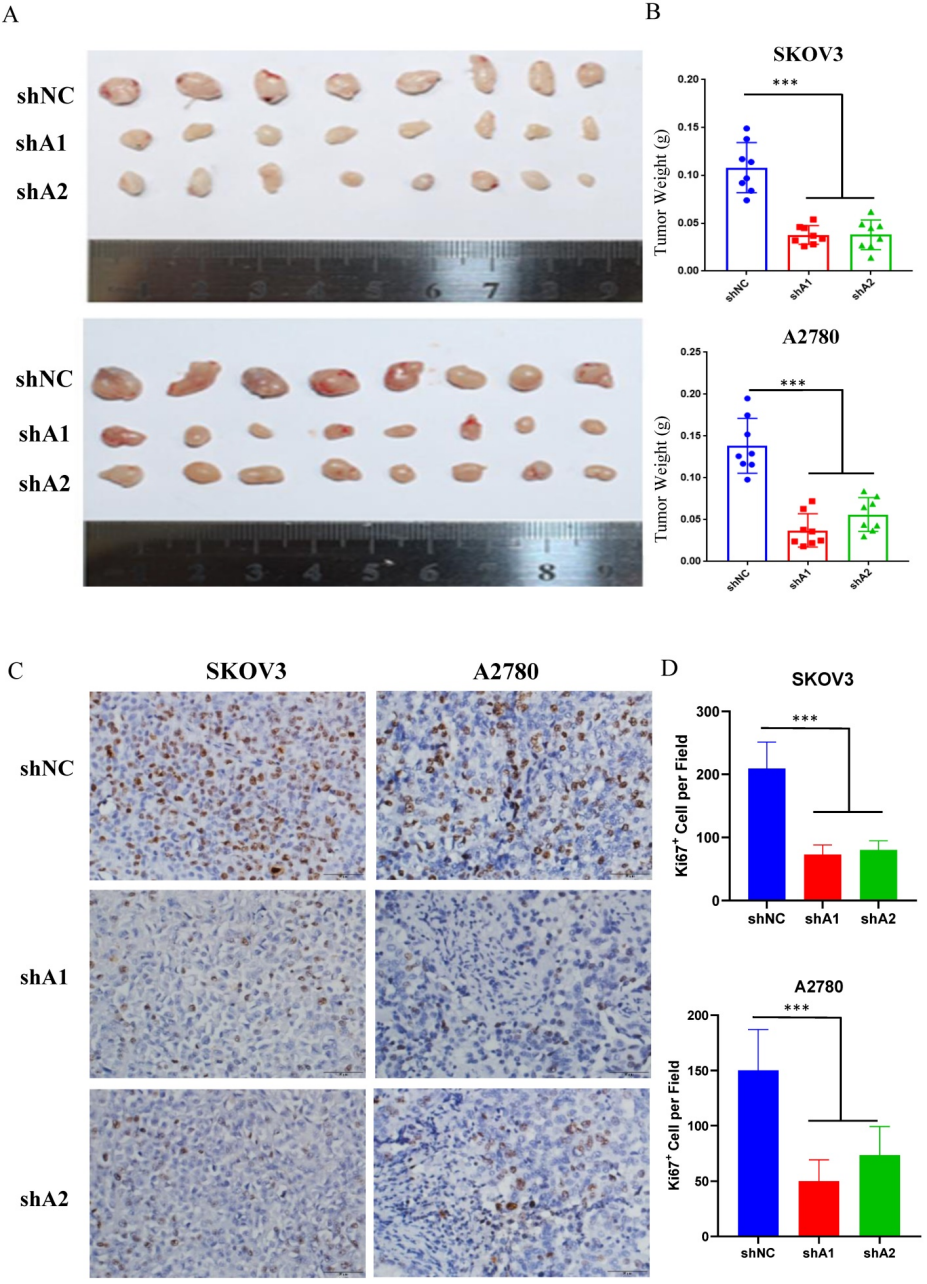
** ADAR1 deficiency represses ovarian cancer tumorigenesis *in vivo*. (A)** SKOV3 and A2780 cells with or without ADAR1 silence were subcutaneously inoculated in BALB/c nude mice. Two weeks later, xenograft tumors were harvested and displayed. **(B)** The weight of xenograft tumors was measured and shown in histograms. Data were shown as means ± SD (***, *P* < 0.001; **, *P* < 0.01; *, *P* < 0.05).

Xenograft engraftment of ovarian cancer cells (SKOV3 and A2780) was generated to analyze the effect of ADAR1 on tumorigenesis* in vivo*. SKOV3 and A2780 cells with or without ADAR1 knockdown were implanted into the right flank of nude mice to generate xenografts. The tumors were collected for further analysis after 14 days. As shown in Fig. 3a, b, ADAR1 shRNA-treated SKOV3 and A2780 cells showed significantly lower tumorigenicity compared with scramble shRNA-treated cells, reflected by smaller tumor volume and lower tumor weight. Further immunohistochemistry staining of the proliferation marker Ki67 indicated that the number of Ki-67 positive cells was significantly reduced in ADAR1 silence group, compared with control group (Fig. 3 c, d).

### Loss of ADAR1 impairs ovarian cancer normal cell cycle

**Figure 4:**
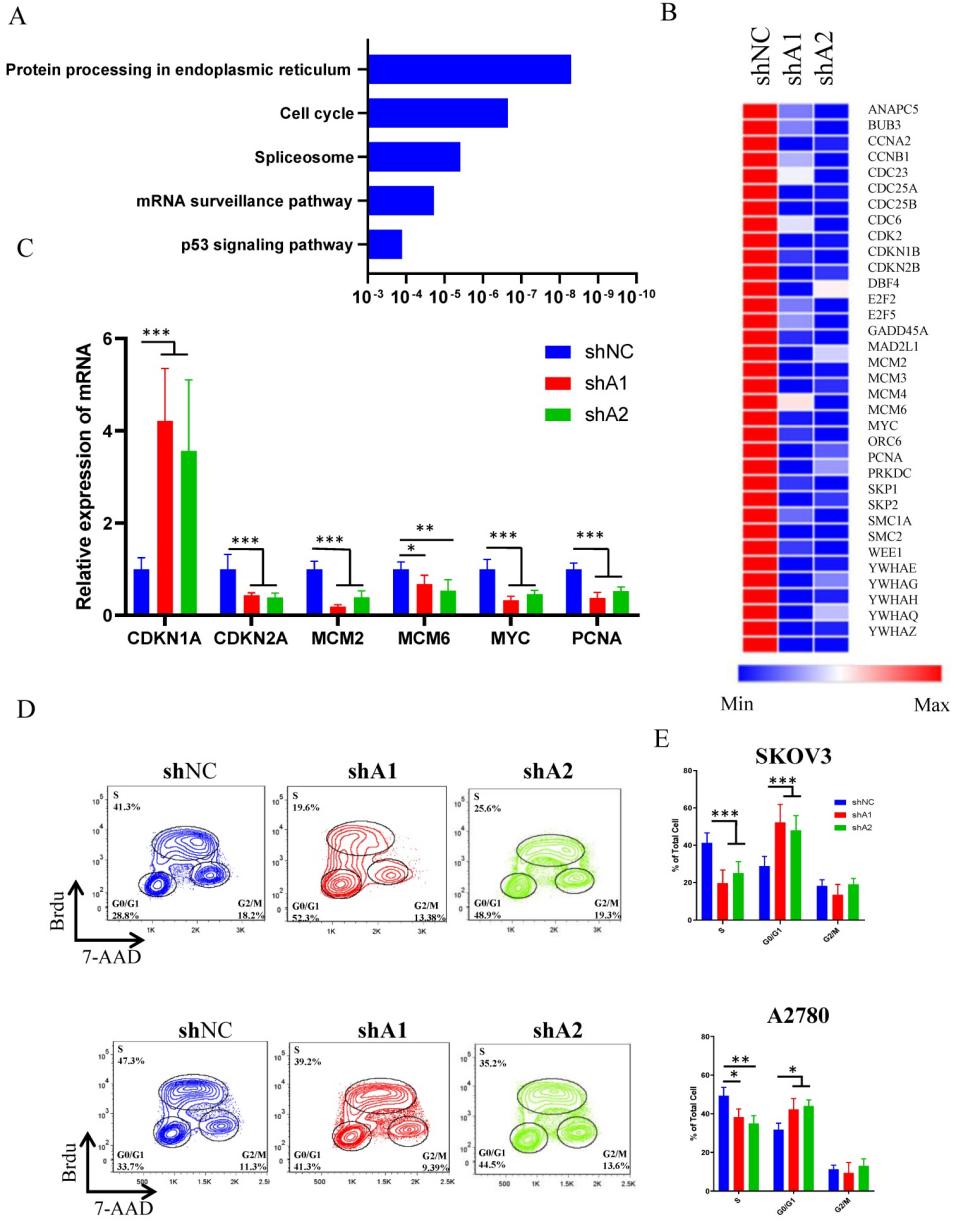
** ADAR1 deficiency causes ovarian cancer cell cycle arrest. (A)** KEGG analysis of differentially expressed genes when ADAR1 deficiency. **(B)** Heatmap of differentially expressed genes in cell cycle pathway when ADAR1 deficiency. **(C)** mRNA levels of genes in cell cycle pathway quantified by quantitative reverse-transcription PCR. **(D)** The representative scatter plot graph showed the cell cycle of SKOV3 and A2780 cells with or without ADAR1 silence. **(E)** Cell cycles in (D) were shown in histograms. All data were shown as mean ± SD from three independent experiments (***, *P* < 0.001; **, *P* < 0.01; *, *P* < 0.05).

To explore the landscape of transcription changes downstream of ADAR1 knockdown, we performed RNA sequencing (RNA-seq) analysis in SKOV3 cells treated with scramble shRNA or ADAR1 shRNA. Our results revealed that knockdown of ADAR1 led to 455 upregulated genes and 1044 downregulated genes (p < 0.05, fold change > 1.5) (Fig. S1, Table S2). The KEGG pathway analysis results revealed that the differentially expressed genes were enriched in protein processing of endoplasmic reticulum, cell-cycle progression, splicing, mRNA surveillance pathway (Fig. 4a), indicating a role of ADAR1 in cell cycle regulation. Moreover, heatmap analysis of cell cycle pathway revealed that the genes involved in the regulation of CMG complex (MCM2, MCM3, MCM5 and MCM6) and cell division cycle (CCNA2, CDC23, CDC25A and CDC25B) were repressed significantly (Fig. 4b, Table S2), and real-time PCR assay confirmed these RNA-seq findings (Fig. 4c). To further demonstrate a role of ADAR1 in cell cycle regulation, BrdU-based cell cycle assay was performed in SKOV3 and A2780 cells. Results revealed that knockdown of ADAR1 led to a reduction in the number of S-phase cells, and an increase in the number of G1-phase cells (Fig. 4d, e), suggesting that silence of ADAR1 caused cell cycle arrest in G1/G0 phase. Taken together, these results indicated that ADAR1 played a crucial role in cell-cycle regulation of ovarian cancer cells.

### Loss of ADAR1 causes ATR-Chk1 mediated DNA damage checkpoint

**Figure 5:**
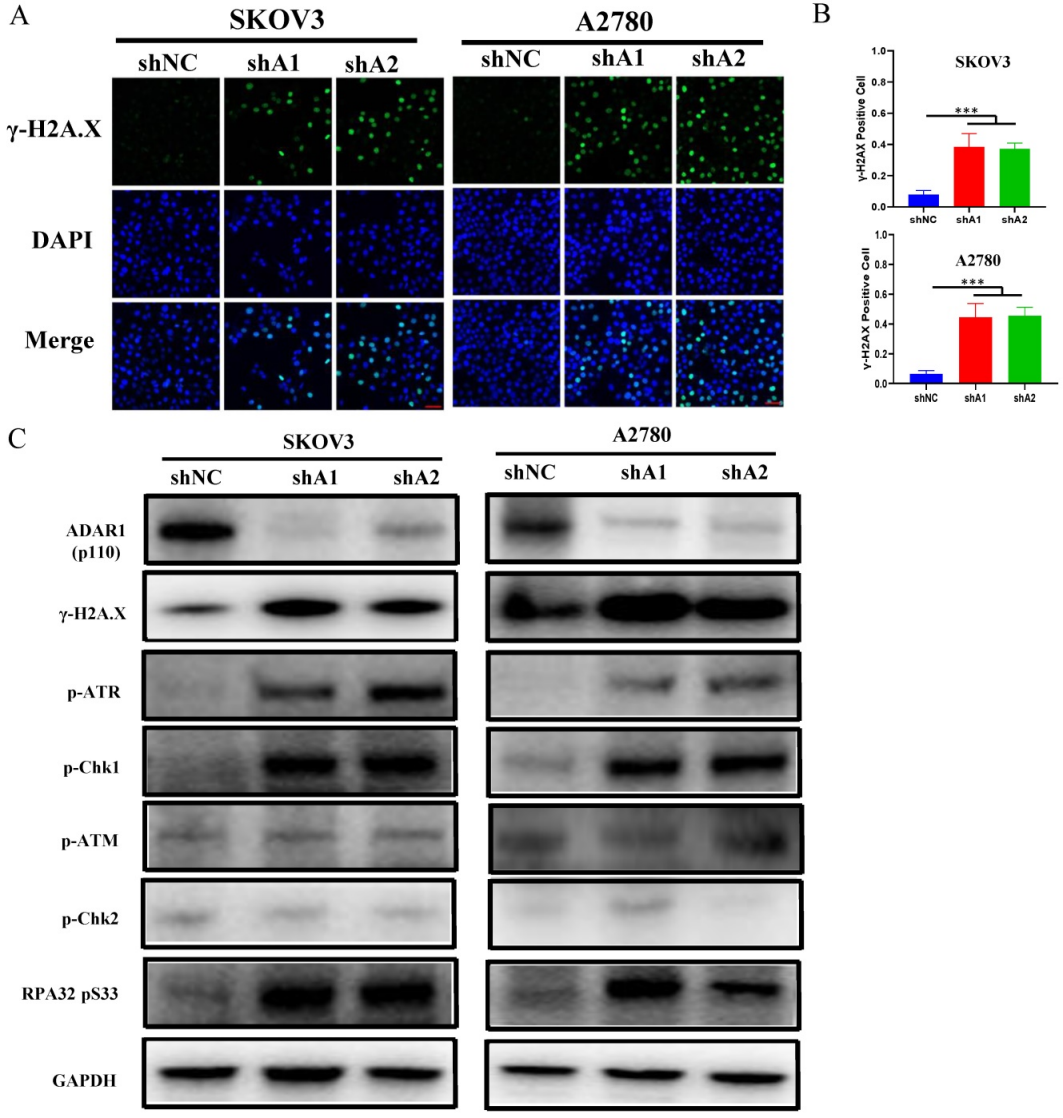
** ADAR1 deficiency cause DNA damage and ATR/Chk1 activation. (A)** The representative microphotographs showed immunostaining of γH2AX foci in SKOV3 and A2780 cells with or without ADAR1 silence. Scale bar, 50 µm. **(B)** Quantification analysis of percentage of γH2AX foci positive cell in (A). All data were shown as mean ± SD (***, *P* < 0.001; **, *P* < 0.01; *, *P* < 0.05). **(C)** Protein levels of DNA Damage Response pathway (phosphorylation of ATM/Chk1, phosphorylation of ATR/Chk2, phosphorylation of RPA32 and γH2AX) were measured in SKOV3 and A2780 cells with or without ADAR1 silence by Western blot.

Loss of ADAR1 has been reported to trigger endogenous type 1 IFN response or PRK activation, thereby repressing cell growth and promoting cell apoptosis in several types of cancer cell lines 15, 18. Surprisingly, we did not observe the activation of endogenous type 1 IFN pathway and PKR when ADAR1 was knocked down (Fig. S2a, b), indicating that ADAR1 silence-induced cell cycle arrest was independent of type 1 IFN or PKR pathway in ovarian cancer cells. Since the RNA-seq results had shown that cell cycle pathway were affected by loss of ADAR1, we next assessed DNA damage status that is critical for cell cycle checkpoint and p53 pathway activation. As shown in Fig. 5a, b, the number of γH2AX foci (a biomarker for DNA damage) was increased when ADAR1 was knocked down in both SKOV3 and A2780 cells.

Previous studies reported that single-strand DNA (ssDNA) generated by replication arrest or DNA damage only cause the phosphorylation of ATR-Chk1, but not ATM-Chk2 19, 20. While double-strand DNA (dsDNA) break could cause the phosphorylation of both ATR-Chk1 and ATM-Chk2. We further detected the phosphorylation status of Chk1, Chk2, ATR, ATM and RPA32. As shown in Fig. 5c, d, loss of ADAR1 only increased the phosphorylation of Chk1, ATR and RPA32, but had no effect on the phosphorylation of Chk2 and ATM. These results indicated that ADAR1 silence activated ATR-Chk1 mediated DNA damage checkpoint in ovarian cancer cells.

### R loop accumulation is involved in ATR-Chk1 activation caused by loss of ADAR1

**Figure 6:**
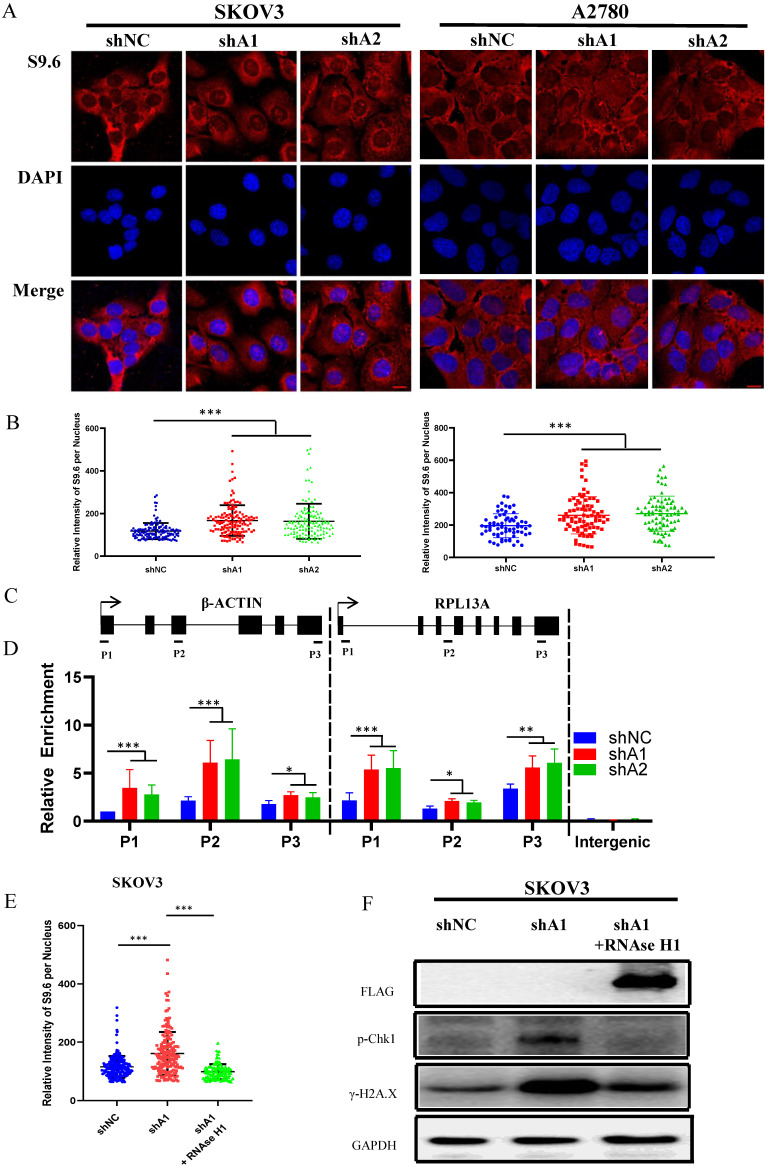
** R loop is involved in ATR/Chk1 pathway activation by ADAR1 deficiency. (A)** The representative microphotographs showed immunostaining of DNA/RNA hybrid (S9.6 antibody) in SKOV3 and A2780 with or without ADAR1 silence. Scale bar, 20 µm. **(B)** Quantification of mean intensity of DNA/RNA hybrid in (A). All data were shown as mean ± SD in n > 90 cells (***, *P* < 0.001; **, *P* < 0.01; *, *P* < 0.05). **(C)** Diagram of the β-actin and RPL13A locus depicting relative position of primer pairs used for DRIP-qPCR. Exons are depicted as black boxes. **(D)** DRIP-qPCR were performed using primers to the indicated regions of the β-actin and RPL13A in SKOV3 cells with or without ADAR1 silence (***, *P* < 0.001; **, *P* < 0.01; *, *P* < 0.05). **(E)** Quantification of mean intensity of DNA/RNA shRNA were transfected with an empty vector or the RNaseH1 expression plasmid (***, *P* < 0.001; **, *P* < 0.01; *, *P* < 0.05). **(F)** Phosphorylation of Chk1 and H2A.X were detected via Western blot in ADAR1 knockdown cells. ADAR1 knockdown cells were transfected with an empty vector or the RNaseH1 expression plasmid.

Previous studies have demonstrated that abnormal accumulation of R loops can cause activation of ATR-Chk1, but not ATM-Chk2 pathway 19, 21, 22. Hence, we speculated that cell cycle arrest induced by ADAR1 silence might result from R-loops accumulation. To confirm this, we used S9.6 antibody to detect the RNA-DNA hybrid component of R-loops. As shown in Fig. 6a, b and Fig. S3c, d, knockdown of ADAR1 caused a significant increase in RNA-DNA hybrids fluorescent in SKOV3, A2780 and U2OS cells, indicating that ADAR1 silence induced intense R-loop accumulation. To further determine whether R-loops accumulation occurred to specific genes, we performed DNA/RNA immunoprecipitation (DRIP) analysis for two well-studied gene locus (β-actin and rpl13a locus) by real-time PCR 23. Primers for DRIP-qPCR were designed for different regions of the genes, including transcriptional start region, gene body (intron and exon), and transcription termination region (Fig. 6c). Results revealed that knockdown of ADAR1 significantly increased DNA/RNA hybrids in nearly all regions of β-actin and rpl13a gene (Fig. 6d). Lastly, we conducted rescue experiments by overexpressing RNase H1 in ADAR1 shRNA-treated ovarian cancer cells, as RNase H1 is a well-studied enzyme that could effectively eliminate intracellular R loop 19. As expected, RNase H1 overexpression significantly reduced the levels of pChk1 and γH2AX in ADAR1-silienced ovarian cancer cells (Fig. 6e-f). Together, these results indicated that loss of ADAR1 could cause R loop accumulation and thus activated ATR-Chk1 cell cycle checkpoint to repress cell growth and tumorigenesis.

### ADAR1 interacts with DHX9 to regulate R-Loop formation

**Figure 7:**
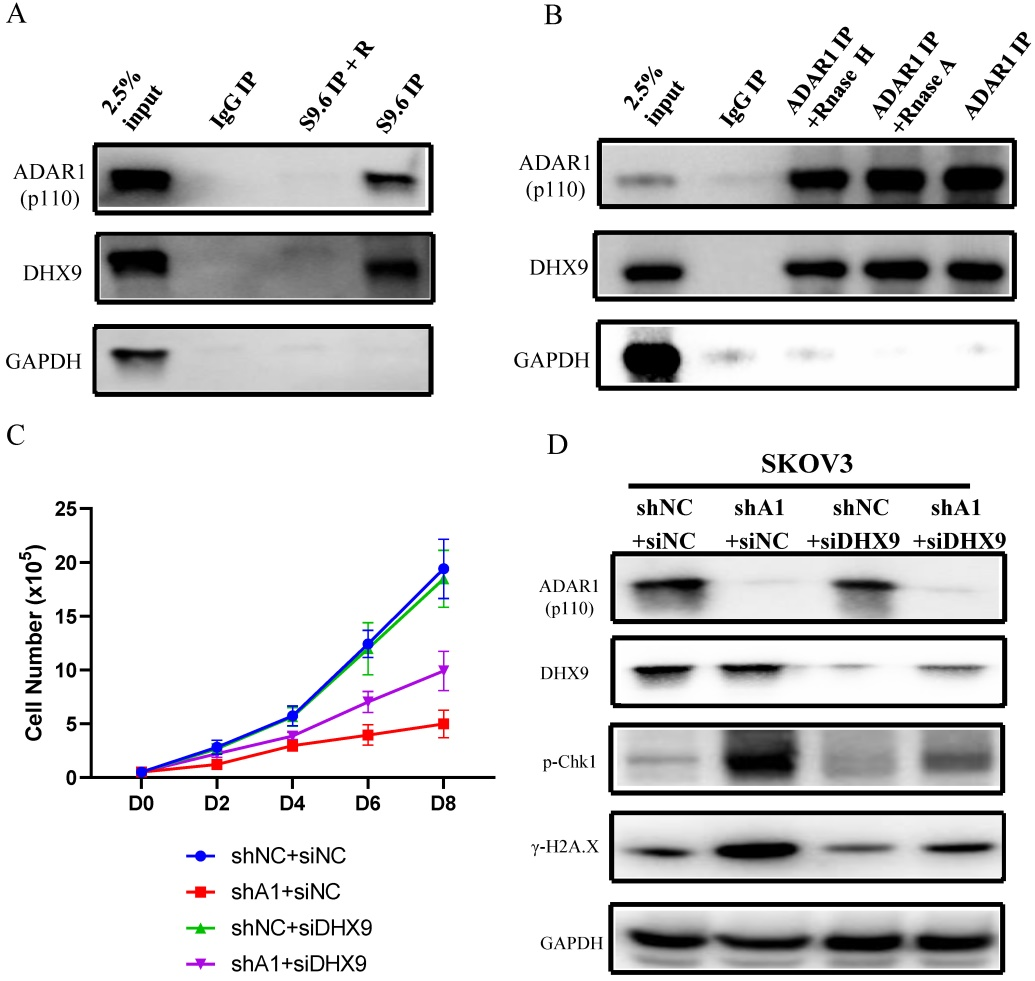
** ADAR1 coupled with DHX9 regulate R-Loop accumulation. (A)** The interaction between ADAR1 and DHX9 was detected by co-immunoprecipitation. **(B)** The interaction between protein and RNA/DNA was detected by DRIP. **(C)** Cell growth curve of SKOV3 with scramble shRNA and SKOV3 and ADAR1shRNA. SKOV3 with ADAR1 shRNA were transfected with DHX9 siRNA or not. **(D)** Phosphorylation of Chk1 and H2A.X were detected via Western blot in ADAR1 knockdown cells. ADAR1 knockdown cells were transfected with DHX9 siRNA or not.

We next investigated the mechanism by which ADAR1 regulates the level of R-loop in ovarian cancer. Firstly, we conducted DNA/RNA-IP experiments by using S9.6 to pull down DNA/RNA hybrid-protein complex. We found that both ADAR1 and DHX9, a well-known ADAR1 interaction protein, were directly interacted with R-loop complex (Fig. 7a), suggesting that ADAR1 might directly regulate R Loop formation. This finding was consistent with two recent reports in which DHX9 has been reported to unwind nascent RNA and facilitate nascent RNA to invade duplex DNA to form R-loop 23, 24. Then Co-IP was performed to confirm previous report of interaction between ADAR1 and DHX9 (Fig. 7b). Of note, RNase A or RNase H treatment had no effect on the interaction, suggesting ADAR1 physical interact with DHX9 independent on RNA or RNA/DNA hybrids. Lastly, to further clarify the role of DHX9, we knocked down DHX9 using siRNA in ADAR1 silenced SKOV3 cells, and found that knockdown of DHX9 could partially improve cell growth and its response to DNA damage in ADAR1-silenced cells (Fig. 7c d). We did not find significantly change of total localization of DHX9 into R loops when ADAR1 knockdown (Fig. S4), suggesting that recruitment of DHX9 into R loops is independent of ADAR1. Taken together, these results indicated that ADAR1 was directly involved in DNA/RNA hybrid-protein complex and interacted with DHX9 to regulate R-loop formation.

### RNA editing regulates R-Loop formation

**Figure 8:**
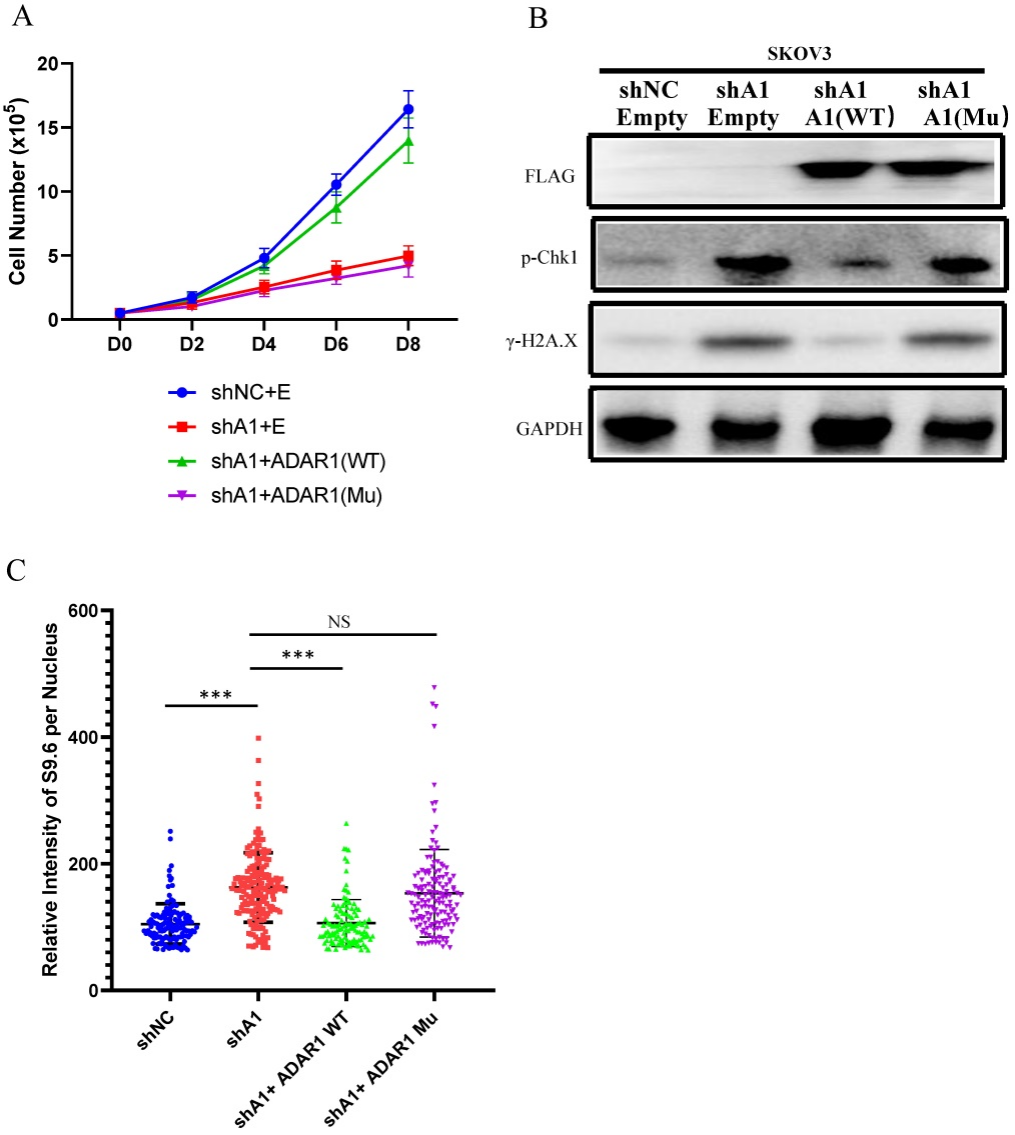
** RNA editing regulate R-Loop formation. (A)** Cells growth curve of SKOV3 with scramble shRNA and SKOV3 with ADAR1shRNA. SKOV3 with ADAR1 shRNA were transfected with human ADAR1 plasmid or human mutation ADAR1. **(B)** Phosphorylation of Chk1 and H2A.X were detected via Western blot in ADAR1 knockdown cells. ADAR1 knockdown cells were transfected. **(C)** Quantification of mean intensity of DNA/RNA hybrid (S9.6 antibody) in SKOV3. All data were shown as mean ± SD in n > 90 cells (***, *P* < 0.001; **, *P* < 0.01; *, *P* < 0.05).

To further explore the mechanism of ADAR1 underlying R-loop regulation, we tested whether RNA adenosine deaminase domain was essential for R-loop regulation. We constructed an ADAR1 expressing vector ADAR1^E912A^ that contained an inactive deaminase domain with E912A mutation (Glu912→Ala912). We overexpressed ADAR1^E912A^ or wild-type ADAR1 in ADAR1-silenced SKOV3 cells. Of note, we found that overexpression of wild-type ADAR1, but not ADAR1^E912A^, could rescue cell growth and DNA damage response in ADAR1-silenced SKOV3 cells (Fig. 8a-b). Similarly, R-loop level was also only rescued by the overexpression of wild-type ADAR1, but not ADAR1^E912A^ (Fig. 8c). Taken together, these results indicated that normal RNA editing was critical for preventing R-loop formation.

## Discussion

ADAR1 has been reported to promote tumor growth and metastasis as an oncogene in several types of human cancers 16, 25-31. In this study, we demonstrated an important role in the tumorigenesis and progression in ovarian cancer. The major findings are as follows: 1) ADAR1 was highly expressed in ovarian cancer tissue and was negatively correlated with progression free survival in ovarian cancer patients; 2) knockdown of ADAR1 suppressed ovarian cancer cell growth and colony formation *in vitro* and inhibited ovarian cancer cell tumorigenesis *in vvo*; 3) cell cycle and transcriptome profile analysis demonstrated that knockdown of ADAR1 induced ovarian cancer cell cycle arrest at G1/G0 stage; 4) loss of ADAR1 disrupted normal dsRNA editing and caused R-loop abnormal accumulation, thereby contributing to ATR-Chk1 pathway activation.

ADAR1 has been shown to act on RNA editing to promote cancer cell survival, growth and migration in different types of cancer cells through multiple molecular functions. For example, in lung cancer, loss of ADAR1 represses cell growth through editing miR-381 25 and inhibits cell migration through editing mRNA of FAK 9. In chronic myeloid leukemia, silence of ADAR1 inhibits leukemia stem cell self-renewal through impairing let-7 miRNA biogenesis 6 or causes cell cycle arrest through repressing miR-26a maturation 29. Recently, it has been reported that deletion of ADAR1 leads to lethal phenotype in cancer cell lines with strong IFN-stimulated gene (ISG) signature through activation of PKR pathway 15, 18. Here, the two ovarian cancer cell lines SKOV3 and A2780 used in this study were negative for ISG signature, as their threshold cycles of IFN-stimulated genes were both more than 35 in real time PCR. Probably due to lack of ISG signature, ADAR1 silence did not elevate PKR activation in SKOV3 and A2780 cells, when ADAR1 was silenced. Together, in ovarian cancer cell lines without ISG signature, indicating that ADAR1 may exert its action in a PKR pathway-independent was in cells without ISG signature.

DNA damage response (DDR) is one of the most important factors leading to p53 activation and cell cycle arrest. When DNA damage occurs, DNA damage-sensing proteins and intermediary proteins will accumulate at the site of DNA damage and activate the conductive protein ATM or ATR 32. Previous studies have shown that double-stranded DNA breaks can directly activate ATM/Chk2 pathway, and also recruit and activate ATR/Chk1 pathway by the single-stranded DNA generated during the repair of double-stranded DNA breaks. However, single-stranded DNA generated by replication arrest or DNA damage can only recruit and activate ATR/Chk1 pathway 20. Our results showed that ADAR1 knockdown caused increase of phosphorylation of Chk1 and ATR, but not phosphorylation of Chk2 and ATM, indicating that single strand DNA was generated by ADAR1 knockdown in ovarian cancer cells. Previous studies revealed that overexpression of ADAR1 caused high edited NEIL1 to enhance DNA damage repair response in multiple myeloma cells and breast cancer cells 33, 34. Perhaps due to low expression of NEIL1 in ovarian cancer, we did not observe a higher DDR in ovarian cancer with ADAR1 overexpression. Together, a proper level of ADAR1 is critical for DDR and cell cycle.

We found that knockdown of ADAR1 caused a global R-loop abnormal accumulation and overexpression of RNase H1 could rescue R-loop accumulation and DNA damage, suggesting that R-loop accumulation plays a key role for DNA damage and cell cycle arrest in ovarian cancer cells. R-loop is a special nucleic acid structure formed during transcription process, which is consisted of two antiparallel DNA strands plus one RNA strand. In this structure, the nascent RNA is base-paired to one of the DNA strands to form DNA/RNA hybrids, while the other DNA strand is unpaired. R-loop has been reported to play an important role in gene transcription 35, immunoglobulin class switch recombination 36 and chromatin epigenetic modification 37. Abnormal R-loop accumulation can lead to replication stress and generate single stranded DNA 21, 22. For response to replication stress and DNA damage, cells can activate ATR pathway that mediates the activation of DNA repair pathways and stabilization of replication forks 38, 39. In ovarian cancer, increased replication stress and DNA damage are prevalent, and inhibition of ATR may represent an effective strategy for ovarian cancer therapy. Recently, a phase 2 clinical trial has shown that administration of the ATR inhibitor berzosertib with gemcitabine increased progression-free survival in patients with high-grade ovarian cancer compared with gemcitabine alone 40, suggesting that ATR inhibitor is beneficial for ovarian cancer therapy. Additionally, myeloid leukemia cells with R-loop accumulation induced by splicing factor disorder has been reported to be more sensitive to ATR inhibitors 21. Hence, it's a good strategy to explore combination ATR inhibitor and ADAR1 inhibitor for ovarian cancer treatment in future studies. Luckily, high-throughput screen of RNA editing inhibitors using a small molecule library has been performed, which could greatly accelerate ADAR1 targeted anti-tumor therapy 41.

DHX9 has been reported to interact with ADAR1 to regulate A to I editing 28 and protect genome from short interspersed nuclear element (SINE) insertions 42. Additionally, DHX9 has been reported to unwind nascent double-stranded RNA during co-transcriptional process, and be implicated in the formation of R-loop 23, 24. Here, we also demonstrated that ADAR1 interacted with DHX9 to directly contribute to the formation of R-Loop complex, which was further supported by our data that silence of DHX9 partly rescued R-loop accumulation resulting from ADAR1 knockdown.

Previous studies have shown that A to I RNA editing by ADAR1 induces various functional changes, such as direct changes in the protein sequence by editing coding region of mRNA 9, 43, changes in miRNA targets by editing pri-miRNA and pre-miRNA target sites 6, 29, altered immunogenicity of endogenous RNA by editing double strand RNA 14, 44, and enhanced mRNA stability by editing 5'UTR region of mRNA 13, 45. Here, our data that only wild-type ADAR1 but not ADAR1^E912A^ could rescue R-loop accumulation and DNA damage suggest that A to I RNA editing is crucial for maintaining R-loop level. However, a global view of RNA editing function on R-loop is still lacking. In future studies, genome-wide methods, including Nascent RNA sequencing and DRIP-Seq should be performed to reveal the accurate relationship between RNA editing and R-loop.

In summary, our work has demonstrated ADAR1 as a pivotal oncogene in ovarian cancer cells. ADAR1 silence induces cell cycle arrest and single strand DNA damage by activating R-loop-driven ATR Pathway. Mechanistically, without ADAR1, low edited double-stranded nascent RNA is unwound by DHX9 to generate free single strand RNA, and then annealing with template DNA strand to generate a strong DNA-RNA hybrid with perfect matched. The novel ADAR1/R-loop/ATR axis is critical for ovarian cancer progression and a potential target for ovarian cancer therapy.

## Materials and methods

### Patients and specimens

Serous epithelial ovarian cancer tissues were obtained from patients who were subjected to surgical resection in the department of Gynecology & Obstetrics, Daping Hospital, Army Medical University (Chongqing, China) between 2015 and 2018. No antitumor treatment was given before the surgery. Fresh ovarian surface epithelium brushings were obtained from the normal ovaries of donors during surgery for other benign gynecological diseases at Daping hospital between 2015 and 2018. All samples were frozen into liquid nitrogen within 10 min after surgical resection and stored at -80 °C until analyses. Tissue array blocks containing ovarian cancer and normal ovarian tissues were established using a tissue microarrayer (Leica, Germany). Procedures for the collection of human samples and their usage for tissue arrays were approved by the Ethical Committee of the Army Medical University (Chongqing, China). All patients signed an informed consent for participating in this study.

### Cell Culture

HEK293T, A2780, IGROV1 and SKOV3 were purchased from Procell Life Science & Technology Co., Ltd (Wuhan, China). All cells were identified by short tandem repeat (STR) profiling and cultured according to the manufacturer's specifications for less than 3 months. HEK293T, A2780 and IGROV1 cells were cultured in Dulbecco's modified Eagle's medium (Gibco, China) containing 10% fetal bovine serum (Gibco, USA) and 1% penicillin (Gibco, USA). SKOV3 and U2OS cells were cultured in McCoy's 5A medium (Gibco, China) containing 10% fetal bovine serum and 1% penicillin. All cell lines were tested mycoplasma negative and cultured in an incubator (Thermo Fisher, USA) gassed with 5% CO_2_ at 37 °C.

### Plasmid Construction and Lentiviral Packing

pLKO.1 vector (Addgene, USA) was used for ADAR1 short hairpin RNA targeted ADAR1 and the negative control with non-target. pcDNA3.1(+) (Invitrogen, USA) vector was used for ADAR1 overexpression. ADAR1 with E912A mutation was generated using Mut Express MultiS Fast Mutagenesis Kit (Vazyme, China). The lentivirus was generated following the manufacturer's instructions. Briefly, pLKO.1 vector, psPAX2 and pMD2.G plasmids (Addgene, USA) were co-transfected into HEK293T cells using Lipofectamine^TM^ 2000 (Invitrogen, USA). Viral supernatants were collected and filtrated using 0.45 um filter. The lentivirus was used immediately or stored at -80 °C. Target cells were infected by lentivirus with 8 μg/ml polybrene (Sigma, USA) and then selected by 2 μg/ml puromycin (Corning, USA) for 5 days to gain the stable cell line. The related sequences of shRNAs and PCR primer were shown in Supplementary Table 1.

### Cell Proliferation and Colony Formation

For cell proliferation assay, SKOV3, A2780 and IGROV1 cells were seeded at 10000 cells/well into 6-well plates. Cell number was counted by TC10 Automated Cell Counter in every two days. Growth curves were plotted using data from three independent biological replicates. For colony formation assay, SKOV3, A2780 and IGROV1 cells were seeded at 500 cells/well into 6-well plates, and then cultured for 14 days in medium containing 10% FBS at 37 °C. The colonies were fixed with methanol and stained with 0.5% crystal violet. The number of colonies were counted through an inverted microscope.

### Cell Migration Assay

A total of 40,000 SKOV3 cells or 80,000 A2780 cells were used for experiment respectively. Cell suspensions (300 µl) without FBS were plated in the upper non-matrigel-coated chamber, and fresh medium containing 10% FBS was added into the lower chamber. After 24 hours of incubation, the cells acrossing the 8 mm size hole were fixed by incubation with methyl alcohol for 15 min and then stained with crystal violet. Microscopic images were randomly taken under a microscopy and the mean number of migrated cells in each well was calculated from at least three random fields.

### Cell Cycle Assay

BrdU based cell cycle analysis was performed using BrdU Flow Kit (eBiosciences, BD, USA) according to the manufacturer's instructions. Briefly, SKOV3 and A2780 cells were cultured for 30 min with 10 μM BrdU. The cells were fixed, permeabilized and then treated with DNase to expose incorporated BrdU. After incubation with anti-BrdU antibodies and 7-AAD, and the cells were subjected to flow cytometer analysis (CytoFLEX, Beckman,USA). Cell-cycle distribution was analyzed using FlowJo software (FlowJo).

### RNA Isolation and Quantitative real-time PCR

For gene expression analysis, total RNA was isolated from cells using RNeasy Mini Kit (Qiagen, German) and reverse-transcribed to complementary DNA using PrimeScript™ RT-PCR Kit (Takara, China). Quantitative real-time PCR (qRT-PCR) was performed using SYBR® Green Master Mix (Bio-Rad, USA). Individual values were normalized to house-keeping gene GAPDH that served as an internal control. Relative mRNA expression levels were calculated using the 2^-ΔΔCT^ method. Primers used in RT-qPCR were listed in Supplementary Table S1.

### RNA Sequencing (RNA-seq) and Bioinformatic Analysis

A total amount of 2 μg RNA per sample was used as input material for the RNA sample preparations. Sequencing libraries were generated using VAHTSTM mRNA-seq V2 Library Prep Kit for Illumina® (Vazyme, China) following manufacturer's recommendations and index codes were added to attribute sequences to each sample. Paired-end sequencing of the library was performed on the HiSeq XTen sequencers (Illumina, San Diego, CA). FastQC was used for evaluating the quality of sequenced data. Raw reads were filtered by Trimmomatic and the remaining clean data was used for further analysis. Clean reads were mapped to the hg38 reference genome by HISAT2 with default parameters. Gene expression values of the transcripts were computed by StringTie. DESeq2 was used to determine differentially expressed genes (DEGs) between two samples. Genes were considered as significant differentially expressed if q-value <0.001 and |FoldChange| >1.5. When the normalized expression of a gene was zero between two samples, its expression value was adjusted to 0.01 (as 0 cannot be plotted on a log plot). Gene expression differences were visualized by scatter plot and MA plot. KEGG pathway analysis identified significantly enriched metabolic pathways or signal transduction pathways enriched in DEGs compared to a reference gene background, using the hypergeometric test. KEGG pathway with false discovery rate (q-value) < 0.05 were considered as significantly altered. Morpheus was used to visualize heat map of DEGs (https://software.broadinstitute.org/morpheus).

### Western Blot Analysis

Cellular proteins were extracted using SDS lysis buffer, separated by 10% SDS-polyacrylamide gel electrophoresis, transferred onto polyvinylidene difluoride membranes and then blocked with 5% defatted milk. After incubation with indicated primary antibodies (1:1000 dilution) at 4 °C overnight and anti-goat or anti-rabbit secondary antibodies (Cell Signaling Technologies, USA) at room temperate for 2 hours, immunoreactive signal was visualized using ECL detection kit (Millipore, USA) according to the manufacturer's instructions.

### Immunofluorescence Staining

The cells were fixed in 4% paraformaldehyde (PFA) for 20 min, blocked with 0.1% Triton X-100 for 1 hours at 25 °C, and then incubated with γ-H2AX antibody (9718, Cell Signaling Technologies, USA) or S9.6 antibody (Kerafast, USA) overnight at 4 °C, followed by Alexa FluorR 488 or 594 goat anti-rabbit IgG secondary antibody (H + L) (Proteintech, China) for 2 hours at room temperate. The nuclei were stained with 300 nM DAPI (Proteintech, China) for 15 min at 25 °C.

### Immunohistochemistry (IHC) Assay

For IHC assays, paraffin-embedded tissue sections were probed with ADAR1 and Ki67 antibodies (1:100) at 4 °C overnight, and then incubated with SignalStain Boost IHC Detection Reagent (horseradish peroxidase, mouse) (Cell Signaling Technologies, USA) at room temperature for 15 min. DAB Horseradish Peroxidase Color Development Kit was used for staining visualization, and hematoxylin was used for tissue counterstain. IHC staining was evaluated using a semiquantitative scoring method. Each sample was graded on the basis of the H-score (H-score = I × P, where I was the staining intensity score and P was the score for the percentage of positively stained cells at each staining intensity level). The I scores were defined as follows: 0, no staining; 1, weak staining; 2, moderate staining; and 3, strong staining. The P scores were defined as follows: 0, <5%; 1, 5-25%; 2, 26-50%; 3, 51-75%; and 4, >75%.

### Immunoprecipitation

Each sample used 5×10^6^ cells, which were lysed in cell lysis buffer (50mM Tris-HCl, 2mM EDTA, 137 mM NaCl, 1% Nonidet P-40). The cell lysates were incubated with indicated antibodies (5 µg) overnight at 4 °C and then incubated with 25 µl protein A/G beads (Thermo Fisher, USA) for 4 h at 4 °C. The beads were washed three times with lysis buffer and immunocomplexes were eluted by 1×SDS loading buffer for 20 min. The collected proteins were transferred to PVDF membrane and immunoblotted with indicated antibodies.

### DNA/RNA Immunoprecipitation (DRIP) and DRIP-qPCR

For DRIP, 10^7^ cells per sample were lysed in cell lysis buffer (50 mM Tris-HCl, 2 mM EDTA, 137 mM NaCl, 0.5% Trixon X-100). The cell lysates were incubated with S9.6 antibody overnight at 4 °C and then with 50 µl protein A/G beads (Thermo Fisher) for 4 h at 4 °C. During incubation, 0.5 ng RNase A (PureLink, Invitrogen) was added to digest RNA when needed. The beads were washed three times with lysis buffer and immunocomplexes were eluted by 1×SDS loading buffer for 20 min. The collected proteins were transferred to PVDF membrane and immunoblotted using proper antibodies.

For DRIP-qPCR, 10^7^ cells per sample were lysed in cell lysis buffer (10 mM Tris-HCl, 1 mM EDTA, 0.5% SDS). Nucleic acid was extracted by phenol/chloroform in phase lock tubes and precipitated by ethanol. Nucleic acid was digested with a combination of restriction enzymes (HindIII, BsrGI, XbaI and SspI) overnight at 37 °C. For RNA/DNA/Protein immunoprecipitation, 5 μg of DNA was incubated with S9.6 antibody (5 μg) in binding buffer (10 mM NaPO4 pH 7.0, 140 mM NaCl, 0.05% Trixon X-100) overnight at 4 °C. Immunocomplexes were incubated with protein A/G magnetic beads (Thermo Fisher, USA) for 2 h at 4 °C. The beads were washed three times with binding buffer and the immunocomplexes were then eluted with elution buffer (50 mM Tris-HCl pH 8.0, 10 mM EDTA, 0.5% SDS) for 45 min at 55 °C. For qPCR, RNA/DNA/protein immunocomplexes were digested with Proteinase K (10 mg/ml) and re-extracted by phenol/chloroform in phase lock tubes and precipitated by ethanol. Quantitative real-time PCR was performed using SYBR® Green Master Mix (Bio-Rad, USA). Primers used for DRIP-qPCR were listed in Supplementary Table S1.

### Animal experiments

A total of 48 12-week-old female BALB/c nude mice were purchased from the Beijing Huafukang BioScience Company. Specific pathogen-free grade facility was employed for mice raising and monitoring. To establish xenograft models of ovarian cancer, a total of 5 × 10^6^ SKOV3 or A2780 cells infected with lentivirus containing shNC, shA1 or shA2 in 100 µl PBS were subcutaneously injected into the right flank of nude mice. After two weeks, all xenograft tumors were harvested, weighed, and fixed in 4% paraformaldehyde. All animal experiments were performed in compliance with the guidelines approved by the Ethics Committee of Medical School of Shenzhen University. Randomization and single blinding were used for measurement.

### Statistical Analysis

Experiments were carried out in three to five technical and biological replicates, and statistical parameters including the sample size (*n*) and the significance analysis were specified in figure legends. GraphPad Prism 6 (La Jolla, CA, USA) was applied for statistical analysis. Two-tailed and unpaired Student's *t*-test were used to calculate significance in an interval of 95% confidence level, following normal distribution with SD values. Data were demonstrated as mean ± SD. *P <* 0.05 was considered statistically significant.

## Supplementary Material

Supplementary figures.

Supplementary table 1.

Supplementary table 2.
