# Supplementary material for: ADAR1 Prevents R-loop Accumulation-Driven ATR Pathway Activation in Ovarian Cancer
Source: J Cancer. 2022 Apr 24;13(8):2397–412. doi: 10.7150/jca.72108 (PMC9174845; doi:10.7150/jca.72108)
Supplement: Supplementary file 1 — Supplementary figures. [file jcav13p2397s1.pdf]

shADAR1 VS shNC

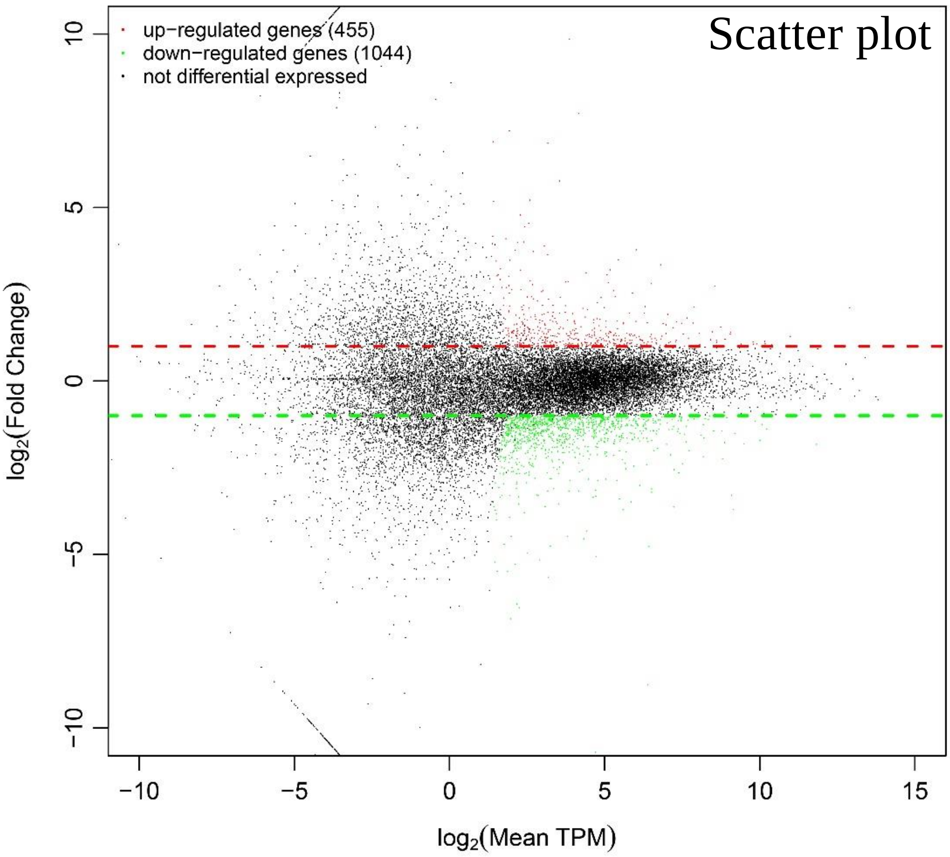

shADAR1 VS shNC

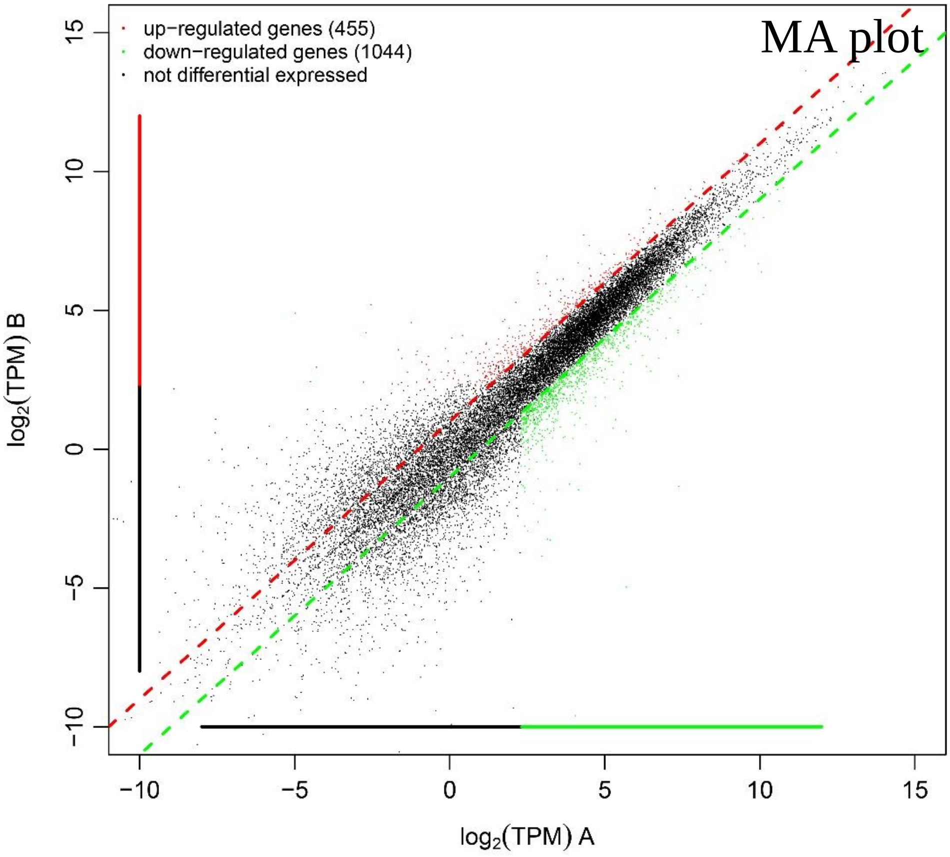

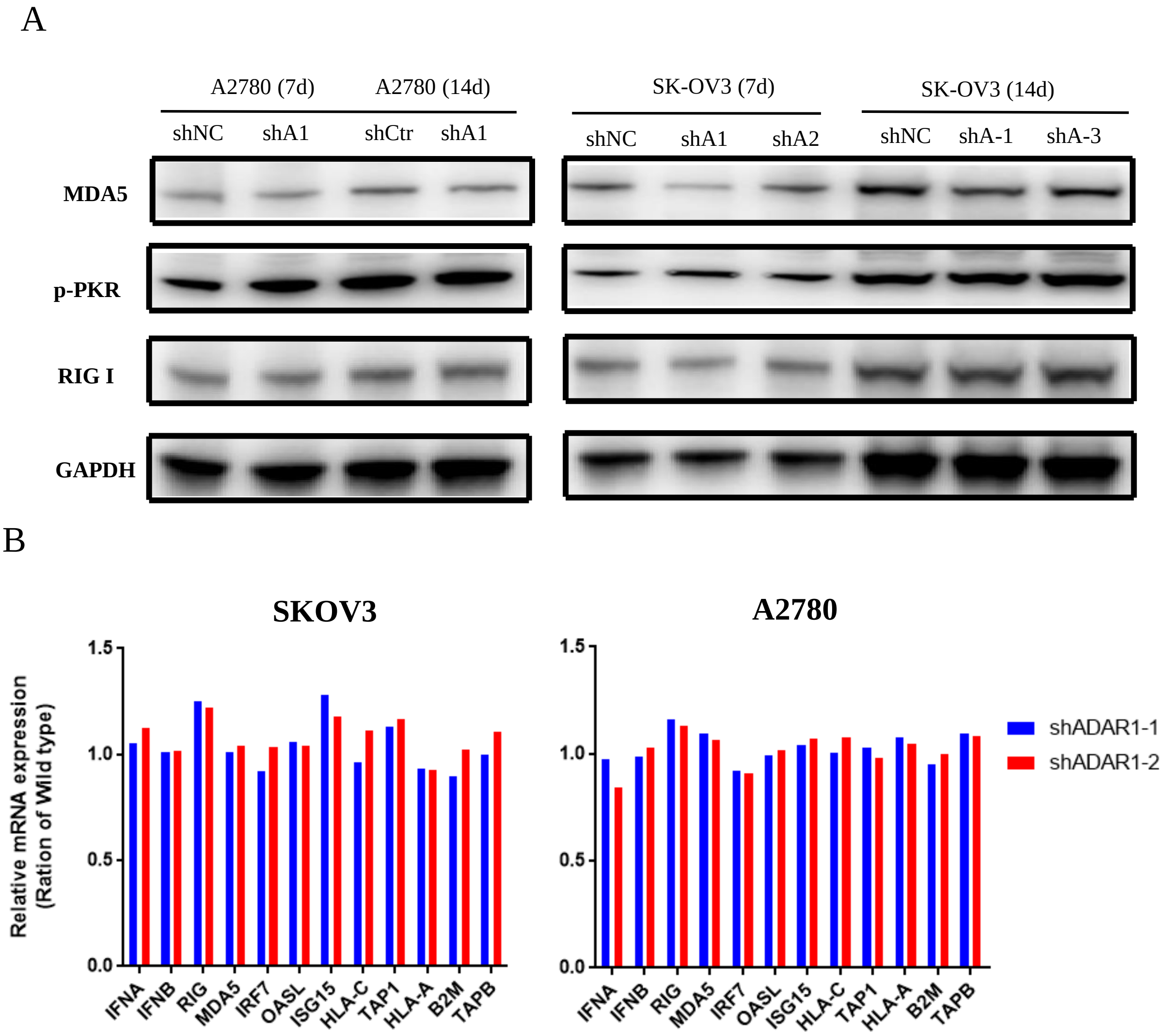
A
A2780 (7d)
A2780 (14d)
SK-OV3 (7d)
SK-OV3 (14d)
shNC
shA1
shCtr
shA1
shNC
shA1
shA2
shNC
shA-1
shA-3
MDA5
p-PKR
RIG I
GAPDH
B
SKOV3
A2780
Relative mRNA expression
(Ration of Wild type)
1.5
1.0
0.5
0.0
IFNA
IFNB
RIG
MDA5
IRF7
OASL
ISG15
HLA-C
TAP1
HLA-A
B2M
TAPB
shADAR1-1
shADAR1-2

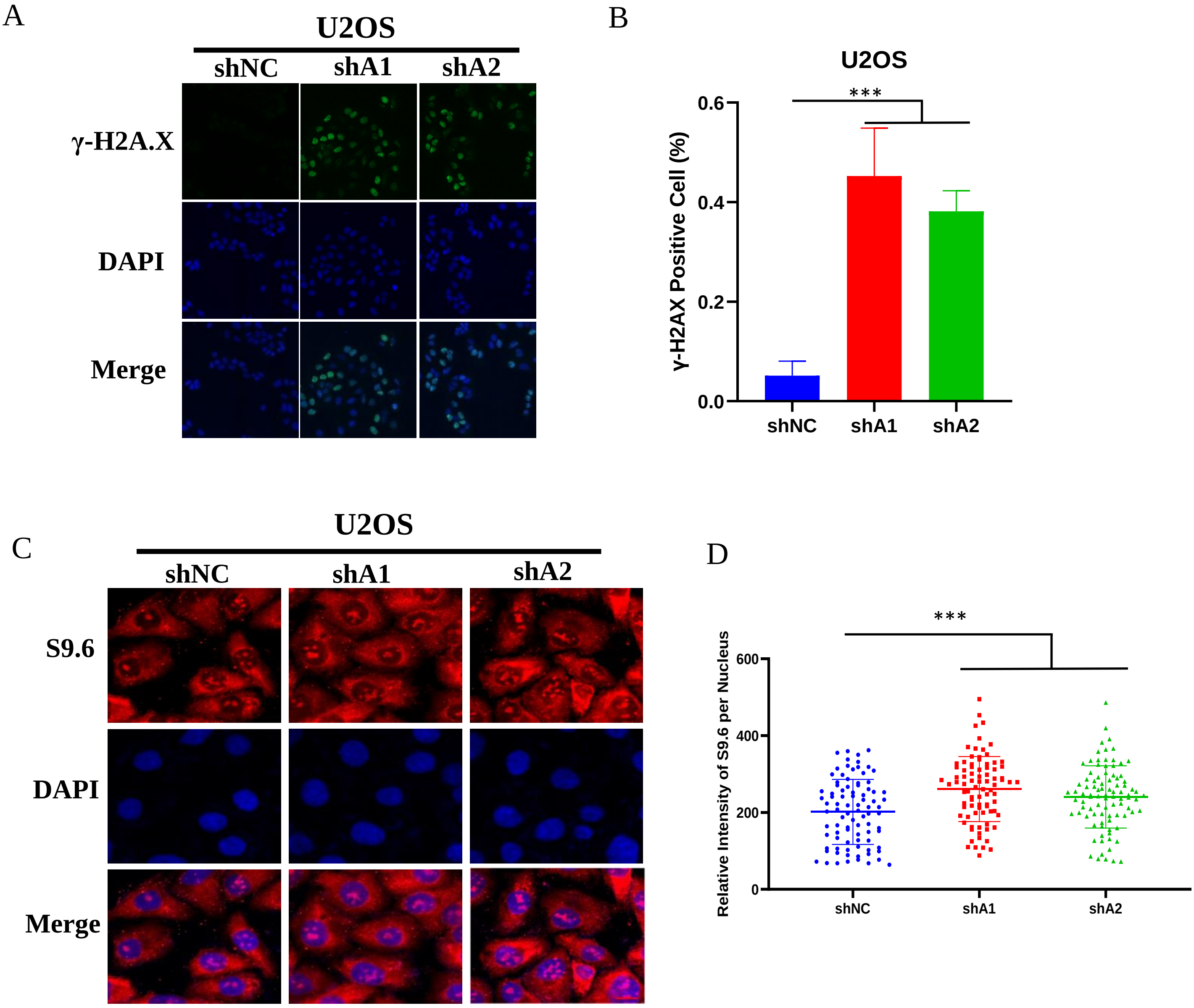
A
U2OS
shNC
shA1
shA2
γ-H2A.X
DAPI
Merge
B
U2OS
***
γ-H2AX Positive Cell (%)
0.6
0.4
0.2
0.0
shNC
shA1
shA2
C
U2OS
shNC
shA1
shA2
S9.6
DAPI
Merge
D
***
Relative Intensity of S9.6 per Nucleus
600
400
200
0
shNC
shA1
shA2

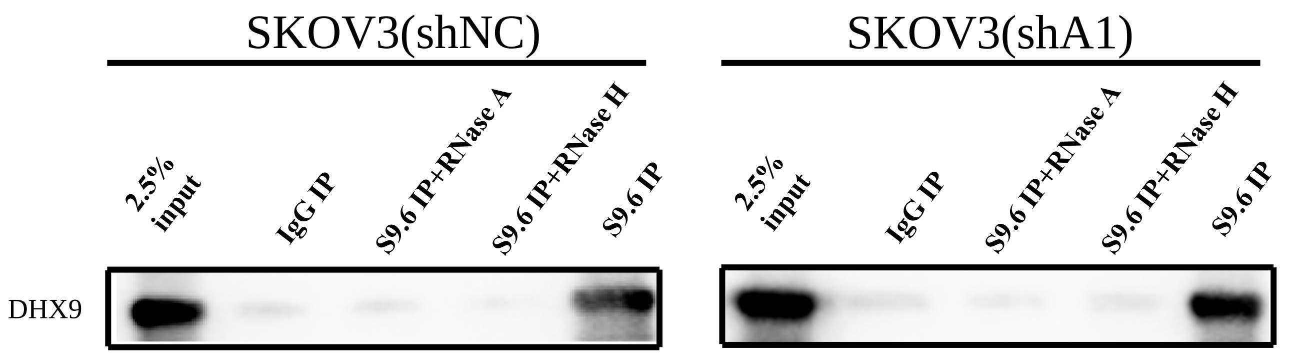
SKOV3(shNC)
SKOV3(shA1)
2.5% input
IgG IP
S9.6 IP+RNase A
S9.6 IP+RNase H
S9.6 IP
2.5% input
IgG IP
S9.6 IP+RNase A
S9.6 IP+RNase H
S9.6 IP
DHX9

Supplement Figure Legends

FigS.1 Differentially expressed genes (DEGs) were visualized by scatter plot and MA plot.

FigS.2 ADAR1 deficiency does not activate type 1 IFN pathway

(A) Protein levels of type 1 IFN pathway (phosphorylation of PKR, MDA5 and RIG1) were measured in SKOV3 and A2780 cells with or without ADAR1 silence by western blot. (B) mRNA levels of type 1 IFN pathway were measured in SKOV3 and A2780 cells with or without ADAR1 silence by qPCR.

FigS.3 ADAR1 deficiency causes DNA damage and R loop accumulation in U2OS cells

(A) The representative microphotographs showed Immunostaining of γH2AX foci in U2OS cells with or without ADAR1 silence. Scale bar, 50 μm. (B) Quantification analysis of percentage of γH2AX foci positive cell in (A). All data were shown as mean ± SD. (***, $P<0.001$ **, $P<0.01$ *, $P<0.05$). (C) The representative microphotographs showed immunostaining of DNA/RNA hybrid (S9.6 antibody) in SKOV3 with or without ADAR1 silence. Scale bar, 20 μm. (D) Quantification of mean intensity of DNA/RNA hybrid in (C). All data were shown as mean ± SD in $n > 90$ cells. (***, $P<0.001$ **, $P<0.01$ *, $P<0.05$).

FigS.4 Recruitment of DHX9 into R loops is independent on ADAR1

The interaction between DHX9 and RNA/DNA was detected by DRIP in SKOV3 with or without ADAR1.
